# Metabolic Syndrome in Older Adults: Through the Lens of Institute for Healthcare Improvement’s (IHI) 4Ms Framework and Social Determinants of Health

**DOI:** 10.3390/life15091370

**Published:** 2025-08-28

**Authors:** Gabrielle Goddard, Shilpa Rajagopal, Gennifer Wahbah Makhoul, Mukaila A. Raji

**Affiliations:** 1 Division of Geriatrics, Department of Internal Medicine, University of Texas Medical Branch at Galveston, 301 University Boulevard, Galveston, TX 77555, USAmuraji@utmb.edu (M.A.R.); 2 John Sealy School of Medicine, University of Texas Medical Branch at Galveston, 301 University Boulevard, Galveston, TX 77555, USA

**Keywords:** metabolic syndrome, social determinants, geriatrics, 4M framework

## Abstract

Metabolic syndrome (MetS)—characterized by dyslipidemia, hypertension, hyperglycemia, and abdominal obesity—is a common, modifiable condition that contributes to functional decline and premature mortality in older adults. The accumulation of MetS components increases the risk of cardiovascular, cerebrovascular, and renal diseases, as well as cognitive impairment and polypharmacy in aging populations. A narrative review was conducted focusing on the management of MetS in adults aged 65 and older. Sources were identified through targeted searches of PubMed and relevant guidelines, with an emphasis on literature discussing geriatric-specific considerations. The review was structured using the Institute for Healthcare Improvement’s (IHI) 4Ms Framework: What Matters, Medication, Mentation, and Mobility. Findings highlight that current MetS guidelines are often extrapolated from younger populations and insufficiently account for geriatric-specific factors such as altered pharmacokinetics, multimorbidity, and social determinants of health. The 4Ms Framework provides a comprehensive lens to adapt these guidelines, supporting individualized treatment plans that consider patient goals, cognitive status, and functional capacity. Incorporating social services and aligning interventions with socioeconomic realities can further bridge disparities in care. The 4Ms framework can help healthcare providers communicate effectively with patients, ensuring treatment plans align with evidence-based practices and the patient’s individual priorities. Treatment of MetS must be tailored to individual patient needs based on presented risk factors, severity of risks, and social determinants of health. Adjusting treatment plans in accordance with the socioeconomic status (SES) of patients will allow for systematic improvement of outcomes.

## 1. Introduction

Metabolic syndrome (MetS), also known as Syndrome X, is a cluster of metabolic abnormalities characterized by insulin resistance, central obesity, and dyslipidemia accompanied with chronic low-grade inflammatory state; these factors increase the risk for the development of chronic diseases, such as cardiovascular disease and type 2 diabetes mellitus (T2DM) [1,2,3,4]. Several definitions with varying diagnostic criteria have been proposed, with the most commonly used being those from the National Cholesterol Education Program (NCEP) Adult Treatment Panel III (ATP III) and the International Diabetes Federation [5].

The prevalence of adult MetS continues to rise globally, and the aging population is not exempt from this. Data from the United States National Health and Nutrition Examination Survey (NHANES) from 2011–2018 demonstrated a significant increase in MetS prevalence among participants, from 37.6% in 2011–2012 rising to 41.8% in 2017–2018 [1,6]. Between 1999 and 2018, there was a significant increase in MetS in patients aged 65 and older from 55.78% to 63.012%, respectively [7]. Among these patients, older adults residing in the South Atlantic, Midwest, and South regions of the United States exhibited higher rates of MetS during this period [8].

Given the rising prevalence of metabolic syndrome (MetS) and the unique challenges associated with aging, there is a pressing need for management strategies tailored to the geriatric population. This review aims to present a comprehensive approach to managing MetS in older adults by integrating the Institute for Healthcare Improvement’s (IHI) 4Ms Framework with consideration of Social Determinants of Health (SDoH). We explore current diagnostic criteria, underlying pathophysiology, key comorbid conditions, and practical applications of the 4Ms in clinical care. Our ultimate goal is to support healthcare providers in promoting early diagnosis, implementing patient-centered strategies, and improving health outcomes for this highly vulnerable population.

## 2. Methodology

### 2.1. Research Model

This paper follows a narrative review model aimed at synthesizing existing literature on metabolic syndrome (MetS) in older adults, with a focus on the application of the Institute for Healthcare Improvement’s (IHI) 4Ms Framework and consideration of the Social Determinants of Health (SDoH). The objective was to integrate evidence from diverse study designs to provide a comprehensive, clinically relevant overview rather than conduct a meta-analysis.

### 2.2. Data Collection and Search Strategy

A comprehensive search of the PubMed database was performed from the inception to February 2025 to identify relevant articles. Various medical subject headings (MeSH) terms, including “geriatric”, “aging”, “4Ms”, “metabolic syndrome”, and “social determinants of health”, were used to generate the search strategy.

### 2.3. Inclusion Criteria

Studies were eligible in this review if they met the criteria:

1. Conducted in older adult populations.
2. Focused on Metabolic Syndrome.
3. Discussed social determinants of health.
4. Included treatments/outcomes.
5. Published in the English language.

### 2.4. Exclusion Criteria

Studies were excluded if:

1. They were not conducted on human subjects.
2. They were not published in the English language.

## 3. Data Extraction and Analysis

Two independent reviewers assessed the titles and abstracts of all identified studies to determine eligibility. The full-text articles of potentially eligible studies were then obtained and reviewed in detail by the same reviewers. Any discrepancies between the 2 reviewers were resolved through discussion and consensus. The data was summarized qualitatively due to the heterogeneity of the included studies.

## 4. Risk Factors and Related Disorders

MetS is associated with multiple risk factors, including various chronic diseases such as dyslipidemia, hypertension, hyperglycemia, central obesity, and non-modifiable risks. Dyslipidemia, defined as abnormal lipid levels in the bloodstream, increases the risk of cardiovascular diseases and can cause inflammation and oxidative stress [9]. For MetS, dyslipidemia is present when triglyceride levels are ≥150 mg/dL and high-density lipoprotein cholesterol is <40 mg/dL in men or <50 mg/dL in women [1]. Hypertension, another key risk factor, refers to chronic elevation of blood pressure that may result in end-organ damage [10]. In the context of MetS, the threshold for blood pressure is ≥130/85 mmHg [1]. Hyperglycemia, which is characterized by elevated glucose levels, can lead to oxidative stress, alter gene expression, and lead to inflammation with threshold levels > 100 mg/dL [1,11]. Central obesity, determined by waist circumference, is abnormal or excessive adiposity accumulation in the abdominal region [12]. While cut-off values may vary by sex and race/ethnicity, central obesity is generally defined as WC ≥ 102 cm in men or ≥88 cm in women [1]. The development of MetS involves the complex interaction of lifestyle, environmental, and genetic factors [3,13]. Metabolic syndrome and related disorders such as obesity, pre-diabetes, T2DM, diabetic microvascular complications, and metabolic dysfunction-associated steatosis liver disease are characterized by inflammation [14,15].

## 5. Diagnostic Criteria

With the different definitions and diagnostic criteria suggested for MetS, difficulties have arisen in the comparison of data from studies, given the use of different criteria. Two major diagnostic criteria are used to define metabolic syndrome. Table 1 shows the 2005 NCEP ATP III, and Table 2 shows the 2009 criteria from the International Diabetes Federation [5,16].

## 6. Pathogenesis of Metabolic Syndrome

Several mechanisms have been proposed for the development of MetS in the general population. These include insulin resistance, a chronic low-grade proinflammatory state, and neurohormonal changes [4,17]. In response to elevated blood glucose levels, pancreatic beta cells secrete insulin, which inhibits lipolysis and hepatic gluconeogenesis while promoting glucose uptake in peripheral organs [18].

With insulin resistance, there is impaired lipolysis in adipocytes, leading to an increase in free fatty acids (FFAs). The accumulation of FFAs drives a paradoxical compensatory hyperinsulinemia in order to maintain euglycemia. Eventually, exhaustion of this mechanism occurs, which in turn leads to a decrease in insulin levels, further exacerbating the lipotoxic effects of FFAs [19]. One such effect is increased organ lipid accumulation, like skeletal muscle, along with increased blood triglyceride levels and the production of very low-density lipoproteins [20]. Various pathways contribute to a cumulative pro-inflammatory state in MetS, with elevated Interleukin-6 (IL-6), C-reactive protein, and tumor necrosis factor alpha (TNFα) among these individuals. IL-6 has been shown to be increased with insulin resistance and obesity, is released by both macrophages and adipocytes responsible for the increased production of acute-phase reactants in the liver, like CRP, and promotes pathways leading to vascular wall atherosclerosis and endothelial dysfunction [21,22]. In previous studies, high CRP demonstrated a correlation with individuals with T2DM and MetS [23]. TNFα, which is produced by macrophages in adipose tissue, contributes to insulin resistance through various mechanisms and impairs adipocyte and hepatocyte insulin signaling, inducing hepatic lipolysis and increasing FFA levels [24,25].

There are different opinions on the causation of the disease, as different risk factors are interrelated and complex [2]. There has been progress in the field in identifying genetic markers of the risk factors, namely obesity and insulin resistance. Rare recessive mutations in the hormone leptin and its receptor and mutations in melanocortin receptors 3 and 4 are associated with monogenic obesity [26]. The constellation of these factors puts patients at a heightened risk for atherosclerotic cardiovascular disease, stroke, myocardial infarction, chronic kidney disease, and fatty liver disease, among others [17].

Individuals of older age have increasing rates of MetS when compared to younger individuals, and MetS itself is also involved in higher rates of aging. Aging is considered a biological process with progressive deterioration of physiological functioning. In MetS, there is a significant excess of reactive oxygen species, which in turn then to contributes to the aging process [27]. In a study by Moore et al., an analysis of the data from the NHANES showed that advanced age was independently associated with an increased likelihood (OR 1.73; 95% CI) of metabolic syndrome during 2007–2012 [28]. However, little is known about how to approach MetS with the 4Ms method or age-friendly management of the different components of MetS [29,30].

## 7. Metabolic Syndrome in Older Adults

Managing the risk factors of MetS is key in avoiding the manifestation of related diseases, primarily cardiovascular disease, and mitigating the onset of disability, poor quality of life (QOL), institutionalization, and premature death in older adults. Treatments that place emphasis on lifestyle changes (e.g., exercise and diet changes) and optimizing other social determinants of health (e.g., education, income, housing, access to quality food, and neighborhood walkability) are especially critical in managing MetS in the geriatric population. This is due to the large impact of suboptimal Social Determinants of Health (SDoH) on functional recovery and access, quality, and outcomes of care in this population [31]. With difficult-to-manage symptoms, an age-friendly pharmacologic therapy regimen may be implemented to manage dyslipidemia. These lifestyle changes can prove challenging to patients of lower SES [17]. In a longitudinal study on MetS remission based on SES with mediated health behaviors, after 3.8 years, 42.7% of participants with MetS experienced remission. Individuals with higher education and income level showed a statistically significantly greater likelihood of remission [32]. When treating older adults with MetS and multimorbidity, healthcare providers should be aware of the impact of SES on treatment outcomes, including health literacy, self-management capacity, perceived barriers to care, and social determinants such as living conditions [32]. A management plan created by providers, patients, and their families that holistically addresses the SdoH, lifestyle, and behavioral drivers of MetS is key to preserving independent living and improving outcomes of care in the geriatric population.

## 8. Adapting IHI 4Ms to Optimize Care of Geriatric Patients with Metabolic Syndrome

**Figure 1:**
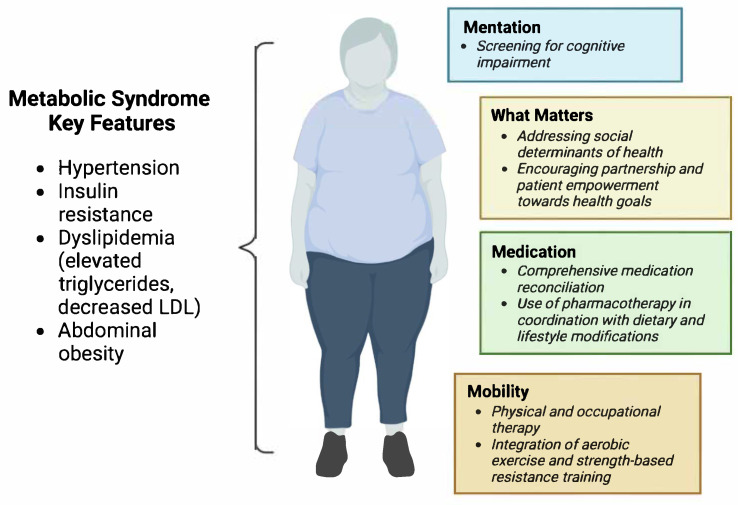
Characteristics of metabolic syndrome in relation to the 4Ms framework. This image was created by the authors of this article. Created in Biorender. Gennifer Wahbah Makhoul. (2025) BioRender.com.

In an effort to address the needs of the growing population of adults aged 65 and older, the Institute for Healthcare Improvement (IHI) created a set of four elements considered critical to high-quality and age-friendly eldercare, termed the “4Ms.” It is defined as What Matters, Medication, Mentation, and Mobility [2,29,33]. The 4Ms—an essential set of evidence-based practices—are part of a broader goal to establish IHI age-friendly health systems that would guide the health system in providing holistic care to older patients [34]. This approach would acknowledge other determinants of health, such as the socioeconomic gradient in healthcare, access to quality care, education, and environment, among other social considerations. The framework emphasizes minimizing harm while aligning care plans with the priorities of the patient and their family and caregivers [34]. However, Yoo et al. found that disparities persist in the application of the 4Ms across racial and ethnic groups [35]. Their retrospective study examined differences in the documentation of the 4Ms during telehealth-based primary care visits by analyzing records from 254 Medicare patients in Southern Nevada [35]. The results revealed significant disparities: documentation of “What Matters” was significantly lower for Asian/Hawaiian/Pacific Islander and Hispanic individuals compared to White and non-Hispanic patients, with adjusted odds ratios of 0.39 and 0.18, respectively [35]. Additionally, Black patients were significantly less likely to have “Mobility” addressed (OR = 0.35) [35]. The findings highlight that mere adoption of the 4Ms framework may not eliminate provider bias in virtual care and call for targeted strategies to ensure equity in geriatric telehealth services. As illustrated in Figure 1, we map the characteristics of metabolic syndrome (MetS) onto the 4Ms framework. Framing MetS through the lens of the 4Ms may enhance care delivery and outcomes by addressing aging-related physiological changes and multimorbidity in a patient-centered manner.

### 8.1. What Matters

As MetS presents as a combination of risk factors for other serious conditions, the management of symptoms is vital for the health of the patient, via a care plan that aligns with the patient’s healthcare priorities and preferences. The treatment of older patients with MetS involves a holistic approach combining lifestyle changes such as activity level, controlling dietary intake, and, if necessary, pharmacotherapy. It is essential for providers to engage patients and their families in a shared decision-making framework, ensuring that therapeutic interventions align with the patient’s abilities, preferences, and willingness to participate. Treatment choices should be driven by conversations about what matters most to the patient and their families. Taking into consideration gender/sex, race, education level, geographic location, and occupation type, among other SdoH factors, will substantially influence patient adherence to treatment plans. Implementing and maintaining a diet for patients of lower socioeconomic status might present challenges, as well as medication and increased activity level adherence. The involvement of social workers is crucial for optimal management, as community-based and government-supported social services may be necessary to address economic barriers and other social determinants of health. These include food insecurity, lack of transportation, and financial assistance for medications. Combining what is important to patients with the effective use of standard treatment options and available community-based resources is key in managing MetS components and their associated multimorbidity and mitigating the onset of disability, frailty, and nursing home placement.

### 8.2. Medication

The management of risk factors of MetS is primarily addressed by increasing exercise levels, adjusting dietary intake, and other positive lifestyle changes. However, medications are often needed for the optimal management of MetS risk factors and associated comorbidities. Pharmacological management to lower LDL Cholesterol, blood glucose, and/or blood pressure must be tailored to avoid compromising the patient’s mobility and mentation, while minimizing polypharmacy and the use of potentially inappropriate medications [17,36,37]. Patient-centered preference should guide the selection of medications, and in Table 3, we describe an overview of possible medication therapies and their indications.

The specific treatment thresholds for the components of metabolic syndrome are as follows: Controlled dyslipidemia with triglycerides of less than 150 mg/dL and HDL cholesterol greater than 40 mg/dL in men or greater than 50 mg/dL in women [1]—providers should utilize statins to control triglycerides and cholesterol and monitor for any side effects [38]; controlled hypertension with values under 130/85 mmHg—there are various types of medications that effectively control hypertension including diuretics, ACE inhibitors, calcium channel blockers, beta blockers, and ARBs, and providers must select one or a combination of therapies that best serve patient goals [39]. Ideally, patients reach a reduced central obesity with a waist circumference of less than 102 cm in men or 88 cm in women [1]. This can be reached through diet and exercise, and in combination with a variety of drugs that can be used to manage weight, including semaglutide, tirzepatide, liraglutide, orlistat, phentermine-topiramate, naltrexone-bupropion, and setmelanotide [40]. Ideal hyperglycemia values are under 100 mg/dL, with variation from patient to patient [1]. It is important for providers to consider frailty when approaching glucose management, and therapeutic choices must aim to avoid both hyper- and hypoglycemia [41].

Patients at especially high risk may benefit from current weight loss drugs or bariatric surgery if it aligns with the care plan approved by both the provider and patient [17]. To avoid polypharmacy in the aging population, a comprehensive medication reconciliation should be completed to identify the most appropriate management plan, with a focus on selecting medications that address multiple symptoms and thus potentially reduce polypharmacy. For example, GLP-1 drugs such as semaglutide and dulaglutide treat diabetes and obesity, exhibit blood pressure and LDL-lowering effects, and have potential benefits for dementia, sleep apnea (a common MetS comorbidity), and osteoporosis [42,43,44].

Medications can also have a deleterious effect in the treatment of MetS and could potentially contribute to the worsened risk factors [45]. β-blockers, anti-psychotics, antiretrovirals, neurotropic agents, and corticosteroids, among others, can exacerbate obesity, which can lead to overall symptom intensity [46,47,48]. Prescribing these potentially harmful medications should first entail an assessment of the risk of symptom aggravation weighed against the positive effect of the medication, especially in the psychiatric realm [45,49]. Elderly patients require monitoring of prescriptions to properly assess the necessity of these medications to reduce inappropriate prescribing and adverse drug effects [50].

There are several barriers to medication accessibility among elderly patients, including medication-related, patient-related, or healthcare-related. In some cases, older adults often misunderstand medication instructions due to polypharmacy, poor literacy, poorer vision, and memory issues [51]. In Slovenia, clinical pharmacists have been integrated into the primary level of healthcare (i.e., family medicine clinics) in order to address these issues [52]. Robust social support is vital for medication adherence, and family and neighbors play a major role. Rural communities tend to have more community-based support, whereas urban settings are often depicted to be more socially isolating [53,54]. To address this, early consultation with social workers is important as they can identify programs and resources in the community to help patients with suboptimal SdoH for financial, transportation, food, and other supports that can help patients adhere to treatment recommendations.

It Is recommended that dietary adjustments be made to the patient’s personal preference and energy needs while ensuring long-term sustainability [55]. The goal is gradual weight loss, targeting a 5–7% weight reduction in individuals with obesity and pre-diabetes [55]. Special emphasis should be placed on calorie reduction, possibly target a lower intake of simple sugars, sodium, cholesterol, trans fats, and saturated fats, while simultaneously increasing the intake of fruits, vegetables, whole grains, and fish [13,17]. Limiting the intake of alcohol, tobacco, and similar substances will also improve outcomes [17]. Dietary changes are unique to the individual, and it is important to address patient priorities in terms of established diets and restrictions based on a variety of factors. Depending on patient comorbidities, healthcare providers should guide patients in making informed nutritional habits with regard to carbohydrate, protein, and fat levels [17]. Food insecurity, medication affordability, and transportation are all barriers to access for patients of lower socioeconomic status. Utilizing food assistance programs and meal delivery services will allow older patients with less access to healthy food options to combat food insecurity. Medication delivery programs, in which medicines can often be delivered in bulk, can be used to help patients with limited mobility have access to their medication and at lower prices.

### 8.3. Mentation

The prevention, identification, and treatment of cognitive issues are necessary in the aging population, especially in the context of MetS [34]. Although there is no definitive consensus in the literature regarding MetS and cognition, one study reported that MetS was associated with poorer brain health, including reduced brain volume, increased vascular pathology, and decreased cognitive performance [56], thus suggesting an association between MetS and the development of dementia. A meta-analysis involving 18,313 participants found that MetS increased the risk of progression from mild cognitive impairment to dementia, with a high incidence of pure vascular dementia [57].

Addressing the cognitive needs of patients requires regular assessments and evaluations to improve overall health outcomes. One medication often used when treating MetS is glucagon-like peptide-1 receptor agonists (GLP1RAs). GLP1RA, developed to treat T2DM, has been found to possess some anti-inflammatory and antioxidant properties, part of the pathogenesis of various diseases [58]. Recent evidence suggests that GLP1RA drugs may play a role in slowing cognitive decline and managing MetS [43,44]. There have been promising studies suggestive of improved cognitive outcomes in dementia patients, particularly in those with cognitive impairment and elevated BMI [59].

Beyond cognitive decline, there is a strong association between psychiatric disorders and MetS [56]. Psychiatric patients face a higher risk of premature all-cause mortality, with the prevalence of MetS estimated at 58% higher in psychiatric patients than in the general population [60,61]. Conversely, poor mental health may also play a role in the behavioral factors—such as physical inactivity or unhealthy dietary habits—that increase obesity risks and worsen health outcomes, especially in older adults [60,62]. Psychiatric diagnoses most commonly associated with MetS are major depressive disorder, bipolar disorder, schizophrenia, anxiety disorder, attention deficit/hyperactivity disorder, and posttraumatic stress disorder [60].

### 8.4. Mobility

Increasing mobility in older patients is vital in improving health outcomes in general. With patients with MetS, it is important to increase physical activity to promote weight reduction and palliate metabolic risk factors [13,17]. For people aged 65 years and older, the WHO recommends 150–300 min of moderate-intensity aerobic exercise or 75 min of high-intensity aerobic exercise per week, or a combination of both [63]. Self-monitoring of activity levels aids in plan compliance [17]. For individuals with MetS, moderate-intensity exercise is recommended, as it particularly helps in reducing body weight and visceral fats and improving blood pressure [64,65]. Such activity also improves insulin sensitivity and increases cardiorespiratory fitness. However, high-intensity training is not recommended, especially in those with cardiovascular diseases [64]. The use of occupational or physical therapists may assist patients with reduced mobility in implementing the necessary lifestyle changes. Evidence suggests that small reductions in body weight improve health outcomes, and providers should prioritize maintaining lower weight levels to promote remission [32]. Safe, daily movement will aid in preserving function and mobility in aging patients, allowing activities of daily living to be performed [34]. Adjusting plans based on what matters to the patient will help providers make informed decisions regarding patient mobility.

## 9. Conclusions

The multiple cardiometabolic factors comprising metabolic syndrome (MetS) are highly prevalent among older adults and represent significant, yet potentially modifiable, contributors to adverse health outcomes. These factors—such as central obesity, dyslipidemia, hypertension, and insulin resistance—often coexist and interact, amplifying the risk of cardiovascular disease, diabetes, and functional decline. This review underscores the importance of approaching treatment recommendations holistically, integrating not only medical management but also the patient’s social, economic, and environmental determinants of health. Effective management requires more than prescribing medications; it demands consistent engagement in lifestyle modifications that are tailored to each individual’s physical capabilities, preferences, and overall health care goals. By aligning interventions with the patient’s priorities and circumstances, clinicians can improve adherence, optimize long-term outcomes, and support healthy aging.

The are several strengths to this review article. First, it uniquely applies the 4Ms framework and offers a geriatric-specific perspective. Second, it effectively highlights barriers that may influence medical management in older adults. Third, it draws on a wide range of recent literature and guidelines. Despite these strengths, it is important to acknowledge some limitations. First, this is a narrative review rather than a systematic review, which may introduce selection bias. Second, much of the evidence for MetS management is derived from studies in younger or mixed-aged populations. Third, disparities exist in the application of the 4Ms framework among clinicians.

## 10. Clinical Implications

For optimal MetS management, we advocate that the care team addresses the multiple medical, functional, and social needs of the older patients through the lens of the IHI 4Ms Framework.

The 4Ms Framework can best be operationalized during annual wellness visits to address patient and family concerns on general health, and also during chronic care management visits.

This framework has been shown to lead to improvement in the process, quality, and outcome of eldercare.

Collaboration with the patients is important in order to address individualized goals, optimize medication use, improve cognitive health, and promote mobility.

## Figures and Tables

**Table 1:** 2005 National Cholesterol Education Program (NCEP) Adult Treatment Panel III (ATP III) diagnostic criteria for metabolic syndrome.

Component	Criterion
Abdominal Obesity	Waist circumference ≥ 102 cm (40 in) in men Waist circumference ≥ 88 cm (35 in) in women
Triglycerides	≥150 mg/dL (1.7 mmol/L) or on drug treatment for elevated triglycerides
HDL Cholesterol	<40 mg/dL (1 mmol/L) in men <50 mg/dL (1.3 mmol/L) in women or on drug treatment for low HDL
Blood Pressure	≥130/85 mmHg or on antihypertensive medication
Fasting Plasma Glucose	≥100 mg/dL (5.6 mmol/L) or on drug treatment for elevated glucose
Diagnosis requires at least 3 out of 5 criteria.	

**Table 2:** The 2009 International Diabetes Federation diagnostic criteria for metabolic syndrome.

Component	Criterion
Waist Circumference	Increased waist circumference with ethnic-specific cut-off values ▪ Europid populations: Males ≥ 94 cm and Females ≥ 80 cm ▪ South Asians populations: Males ≥ 90 cm and Females ≥ 80 cm ▪ Chinese populations: Males ≥ 90 cm and Females ≥ 80 cm ▪ Japanese populations: Males ≥ 90 cm and Females ≥ 80 cm ▪ South and central American populations: Use of South Asian data ▪ Sub-Saharan African populations: Use European data ▪ Eastern Mediterranean and Middle Eastern populations: Use European data
Triglycerides	≥150 mg/dL (1.7 mmol/L) or on drug treatment for elevated triglycerides
HDL Cholesterol	<40 mg/dL (1 mmol/L) in men <50 mg/dL (1.3 mmol/L) in women or on drug treatment for low HDL
Blood Pressure	≥130/85 mmHg or on antihypertensive medication Or treatment for hypertension
Fasting Plasma Glucose	≥100 mg/dL (5.6 mmol/L) or previously diagnosed type 2 diabetes
Diagnosis requires at least 3 out of 5 criteria.	

**Table 3:** Metabolic syndrome treatment algorithm and indications for medications.

Metabolic Syndrome	Indication	Medications
Insulin Resistance	Diabetes Prediabetes	Metformin GLP1RA SGLT2i + Others
Obesity	BMI > 27 kg/m^2^	GLP1RA Naltrexone/Bupropion Orlistat Metabolic Surgery
Hypertension	≥140/90 mmHg (In-office measurement) ≥135/85 mmHg (ambulatory measurements)	ACEi/ARB +CCB +TD +Aldosterone antagonist β-blocker
Dyslipidemia	LDL-C ≥ 70/55 mg/dL (in high risk groups) TG > 200 mg/dL	Statin (maximal tolerated dose) Ezetimibe +Fenofibrate

GLP1RA—Glucagon like peptide-1 receptor agonist; SGLT2i—Sodium-glucose cotransporter-2 inhibitor; ACEi—Angiotensin- converting enzyme inhibitor; ARB—Angiotensin receptor blocker; CCB—Calcium channel blocker; TD—Thiazide diuretic; BMI—body mass index; LDL-C—LDL cholesterol; TG—Triglycerides.

## Data Availability

Not applicable.
